# A Shear-Mode Piezoelectric Heterostructure for Electric Current Sensing in Electric Power Grids

**DOI:** 10.3390/mi10060421

**Published:** 2019-06-23

**Authors:** Wei He, Aichao Yang

**Affiliations:** 1 School of Information Engineering, Baise University, Baise 533000, China; 2 Jiangxi Electric Power Research Institute, Nanchang 330096, China; dkyyac2015@163.com

**Keywords:** piezoelectric current sensing device, two-wire power cord, cymbal structure, force amplification effect, sensitivity

## Abstract

This paper presents a shear-mode piezoelectric current sensing device for two-wire power cords in electric power grids. The piezoelectric heterostructure consists of a cymbal structure and a permalloy plate. The cymbal structure is constructed from a permanent magnet, a brass cap, and shear-mode piezoelectric materials. The permalloy plate concentrates the magnetic field generated by the two-wire power cord on the magnet. Under the force amplification effect of the cymbal structure, the response of the device is improved. A prototype has been fabricated to conduct the experiments. The experimental average sensitivity of the device is 12.74 mV/A in the current range of 1–10 A with a separating distance of d = 0 mm, and the resolution reaches 0.04 A. The accuracy is calculated to be ±0.0177 mV at 1.5 A according to the experimental voltage distribution. The current-to-voltage results demonstrate that the proposed heterostructure can also be used as a magnetoelectric device without bias.

## 1. Introduction

Electricity monitoring of power lines based on current sensing devices is of great significance to improve the security and reliability of the electric power systems. The traditional current sensing devices, such as Hall sensors [1], magnetoresistance sensors [2,3], and current transformers [4], have been investigated. But there are some limitations for these devices. The Hall sensors require external power and place high demands on signal conditioners due to the weak Hall voltage. Magnetoresistance sensors also need external power supply and exhibit large thermal drifts. Current transformers have the disadvantage of magnetic saturation and relay maloperation might be induced. Magnetoelectric (ME) structures based on magnetostrictive materials and piezoelectric materials have been reported for current measuring [5,6,7,8], but the devices need to encircle the wire in operation, which may limit their real applications. Furthermore, most of the previously proposed ME structures require DC bias magnetic field, and the demand for DC bias magnetic field greatly increases the occupied space. Cantilever-based non-invasive piezoelectric current sensors are proposed [9,10,11]. The devices are designed to resonate at the power frequency (50 Hz or 60 Hz) to obtain maximal responses. However, due to the nonlinearity of the piezoelectric materials [12], it is difficult to maintain the resonant state of the devices for varying current amplitudes, and non-resonant structures are more suitable for current sensing. Recently, non-resonant piezoelectric current sensing devices were developed for a single wire in electric power grids [13,14], which operate non-invasively and exhibit high linearity, but the proposed structures are not suited for a two-wire power cord carrying identical currents in opposite directions.

In the past several years, the shear effect of the piezoelectric materials has attracted great attention due to the high piezoelectric constant and electromechanical coupling coefficient [15,16,17,18,19,20,21]. Ren et al. [15] presented a shear-mode piezoelectric vibration energy harvesting device with a maximum output power of 4.16 mW. Carrera et al. [16,17] analyzed multilayered piezoelectric structures in shear-mode using finite element models (e.g., LW models with different theory approximation orders). Liu et al. [21] theoretically investigated the ME effect of a magnetoelectric laminated composite working in shear–shear (S–S) mode, which exhibits stronger ME coupling coefficients. In this paper, a shear-mode non-resonant piezoelectric heterostructure for current sensing of two-wire power cords is designed. The brass cap, the shear-mode piezoelectric materials, and the permanent magnet constitute a cymbal structure, which results in force amplification and potential enhancement of the response to the currents. Theoretical study was conducted, and a prototype was fabricated to investigate the output characteristics of the device. The least squares method is used to analyze the linearity, and the accuracy is investigated for a given electric current. The prototype exhibits high linearity and sensitivity, which are favorable for current sensing in electric power grids. The experimental results verify the theoretical model and the feasibility of the proposed heterostructure. Meanwhile, a conversion factor of 0.77 V/A is obtained for the current-to-voltage conversion experiment, which indicates the latent advantages of the proposed device in practical applications compared with the magnetoelectric laminated composites using DC bias magnetic field [21,22,23,24].

## 2. Structure and Analysis

**Figure 1:**
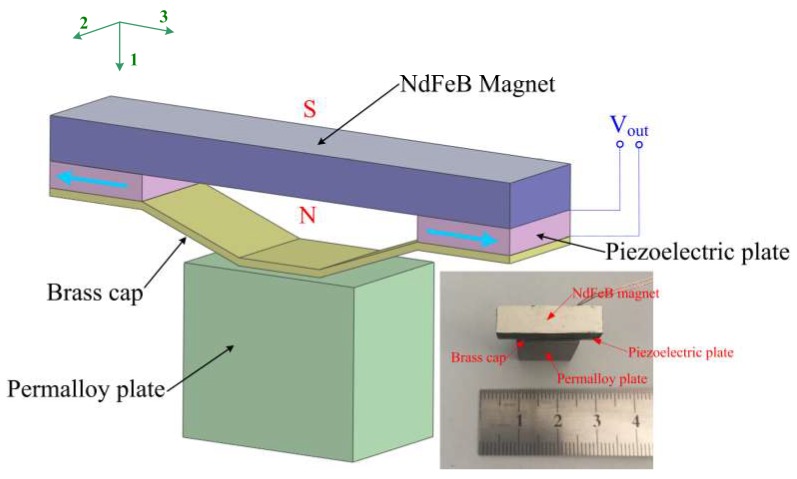
Schematic diagram and photograph of the proposed piezoelectric heterostructure.

Figure 1 shows the schematic diagram and photograph of the proposed piezoelectric heterostructure. The device comprises a cymbal structure and a permalloy plate. The cymbal structure is constructed from a magnet, two shear-mode piezoelectric plates, and a brass cap. The piezoelectric ceramic Pb(Zr,Ti)O_3_ (PZT5H) is chosen as the material of the piezoelectric plates. The dimension of each PZT5H plate is 1 mm (*d_p_*) × 6 mm (*w_p_*) × 3 mm (*l_p_*). The material of the permanent magnet is NdFeB (N35), and the size of the magnet is 5 mm (*d_m_*) × 6 mm (*w_m_*) × 22 mm (*l_m_*). The magnet also acts as a retaining plate for the cymbal structure. Under the action of the AC magnetic field produced by the two-wire power cord, the magnet is acted upon by an AC magnetic force due to the nonuniform AC magnetic field acting on the magnet. The magnetic force results in amplified shear stress on the piezoelectric plates. Then, a voltage is produced, due to the piezoelectric effect of the piezoelectric material.

The magnetic force on the magnet *F_m_* can be expressed as

(1) $$F_{m}=\sigma_{m}\iint_{S_{m}}\Delta HdS_{m},$$

(2) $$\Delta H=H_{1a}-H_{1b},$$

where *σ_m_* is the magnetic charge density, *σ_m_* = *B_r_* [25], *B_r_* is the remnant flux density, *S_m_* is the surface area of one pole of the magnet, and *H*_1*a*_ and *H*_1*b*_ are the magnetic fields on the bottom and top surfaces of the magnet, respectively. *F_m_* can be expressed as a power series of the electric current *I*, and the coefficients of the power series can be determined by fitting curve. Assuming that the vertical force (in 1-direction) acting on one piezoelectric plate by the magnet is *F_m_*/2, the vertical force acting on the magnet by one piezoelectric plate is the same in amplitude and opposite in direction (−*F_m_*/2).

**Figure 2:**
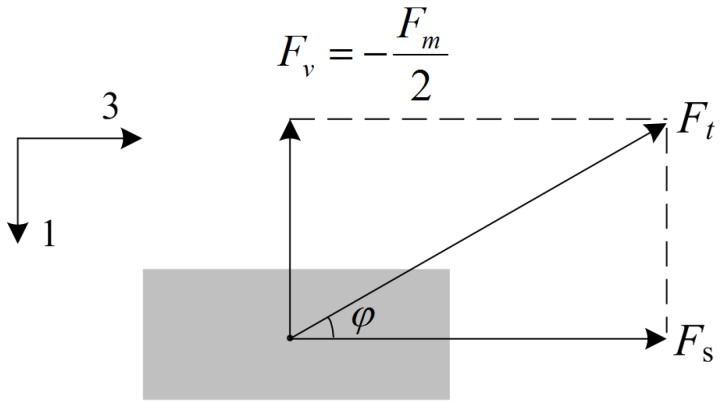
The force *F_t_* exerted by the brass cap on a piezoelectric plate.

Figure 2 shows the force *F_t_* exerted by the brass cap on one piezoelectric plate. Based on the decomposition of the force *F_t_*, the following equation is obtained

(3) $$F_{v}=-\frac{F_{m}}{2}$$

The shear force *F_s_* (in 3-direction) can be expressed as

(4) $$F_{s}=-\frac{F_{v}}{tg\phi },$$

where tgϕ is determined by

(5) $$tg\phi =\frac{2(h-d_{p})}{l_{m}-2l_{p}-l_{t}},$$

where *h* is the distance between the magnet (N pole) and the top part (inner surface) of the brass cap, *d_p_* and *l_p_* are respectively the thickness (in 1-direction) and length (in 3-direction) of one piezoelectric plate, *l_m_* is the length of the magnet (*w_m_* and *d_m_* are respectively the width and the thickness of the magnet), and *l_t_* is the length of the top part of the brass cap. Then, the shear stress acting on one piezoelectric plate is

(6) $$T_{s}=\frac{F_{s}}{w_{p}l_{p}}=-\frac{F_{v}}{w_{p}l_{p}tg\phi },$$

where *w_p_* is the width of one piezoelectric plate. Based on piezoelectric constitutive equations in shear-mode [26], the electric field in 1-direction of the piezoelectric material is given by

(7) $$E_{1}=-h_{15}S_{5}=h_{15}\frac{F_{v}}{w_{p}l_{p}tg\phi c_{55}^{D}},$$

where *h*_15_ is the shear stiffness constant, and $c_{55}^{D}$ is the elastic stiffness coefficient (at constant *D*_1_) in shear-mode. In open-circuit condition, the electric displacement *D*_1_ = 0. Thus, the output voltage of one piezoelectric plate is obtained as

(8) $$V_{1}=E_{1}d_{p}=-\frac{F_{m}h_{15}d_{p}}{2w_{p}l_{p}tg\phi c_{55}^{D}}$$

Based on Equation (8), it is obvious that the voltage is proportional to the magnetic force *F_m_* for determined material and geometric parameters of the device, which depends on the magnetic field magnetic field gradient Δ*H* on the NdFeB magnet. For current-to-voltage conversion application, the conversion factor can be defined as

(9) $$\gamma =\frac{V_{1}}{I_{coil}}=-\frac{F_{m}h_{15}d_{p}}{2w_{p}l_{p}I_{coil}tg\phi c_{55}^{D}}$$

If the output voltage exhibits a linear response to the input current of the coil, the conversion factor *λ* will show a flat response to the current.

## 3. Results and Discussions

**Figure 3:**
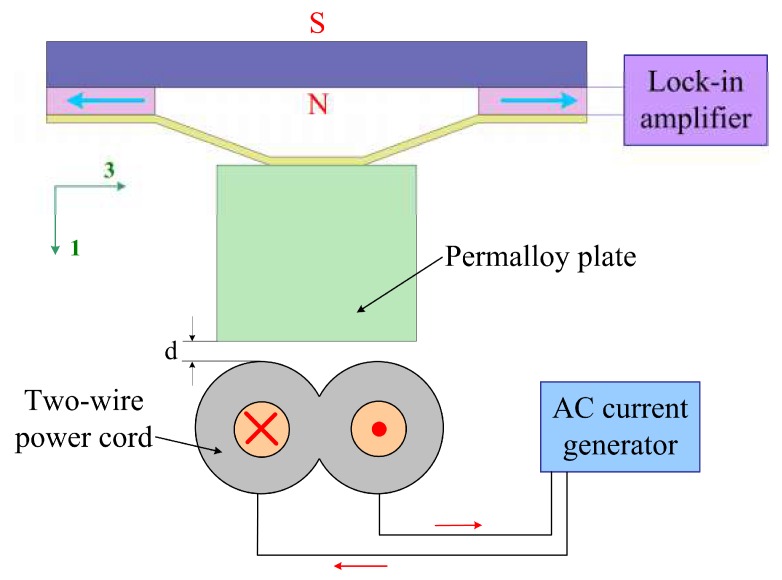
Configuration and experimental set-up for the proposed device.

A prototype was fabricated to study the current sensing performances of the device. The fabrication process is as follows. (1) The brass cap, the piezoelectric plates (PZT5H), the permalloy plate, and the permanent magnet (NdFeB) were dipped in propanone to clean. (2) The brass cap, the piezoelectric plates, and the NdFeB magnet were bonded to constitute the cymbal structure using insulate epoxy adhesive, and the cymbal structure was naturally dried in the air. (3) The permalloy plate was bonded with the cymbal structure. The prototype was then used to current sensing for a two-wire power cord. The configuration and the experimental set-up for the presented device are illustrated in Figure 3 (the power cord carries opposite currents). The electric currents of the two-wire power cord were generated by a current generator, and the output voltages were monitored by a lock-in amplifier. The current sensing device was placed above the two-wire power cord. The permalloy plate concentrates the magnetic field produced by the two wires of the power cord to the NdFeB magnet, which can potentially enhance the response of the device to electric currents. In Figure 3, the parameter *d* represents the distance between the bottom surface of the permalloy plate and the top surface of the power cord.

**Figure 4:**
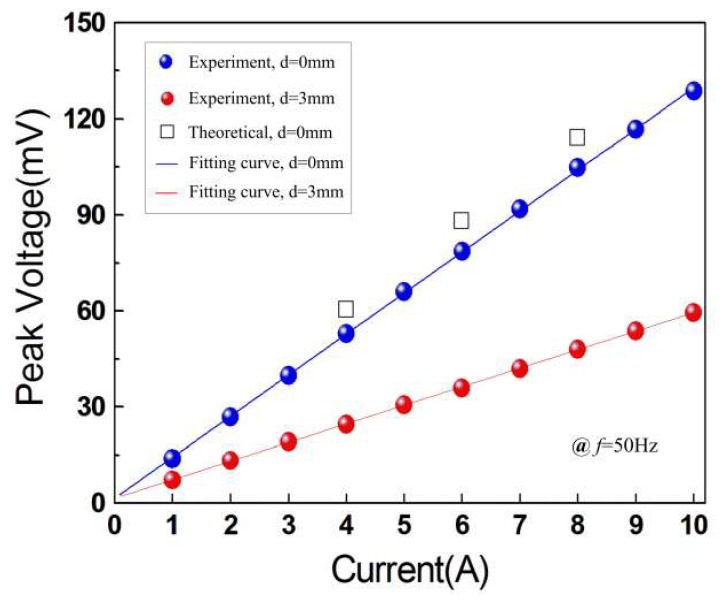
Induced voltage versus the electric current for *d* = 0 mm and *d* = 3 mm.

Figure 4 shows the output peak voltage versus the current in the power cord (*f* = 50 Hz). It can be seen from Figure 4 that the experimental voltage increases from 13.91 mV to 128.59 mV when the current is increased from 1 A to 10 A for *d* = 0 mm, and the average sensitivity is 12.74 mV/A in the given current range (1–10 A). For *d* = 3 mm, there is an obvious drop for the induced voltages, and the voltage varies from 7.23 mV to 59.48 mV with an average sensitivity of 5.81 mV/A. The theoretical voltages obtained from Equation (8) for 4 A, 6 A, and 8 A at *d* = 0 mm were plotted in Figure 4 (respectively 60.5 mV, 88.1 mV, and 114.2 mV), which validate the developed model. In order to analyze the linearity of the proposed device, the least squares method was adopted. The equation of the fitting curve can be expressed as

(10) V=aI+b,

where *a* is the slope and *b* is the intercept of the equation. Using the experimental data in Figure 4, the slope of the equation *a* = 12.82077 for *d* = 0 mm and *a* = 5.785861 for *d* = 3 mm. Correspondingly, the intercept *b* = 1.546273 for *d* = 0 mm and *b* = 1.548193 for *d* = 3 mm. The correlation coefficients are 0.999897 and 0.999945 for *d* = 0 mm and *d* = 3 mm, respectively. After plotting the fitting curves in Figure 4, the linearity of the proposed device is calculated by

(11) $$\delta =\frac{\Delta V_{\max}}{V_{\max}}\times 100\%,$$

where Δ*V*_max_ is maximal deviation between the experimental results and the fitting results, and *V*_max_ represents the full-scale output of the device. The corresponding results are 0.9% and 0.67% for *d* = 0 mm and *d* = 3 mm, respectively. Compared with the resonant structures, the high linearity makes the presented device very suitable for current sensing in electric power systems.

**Figure 5:**
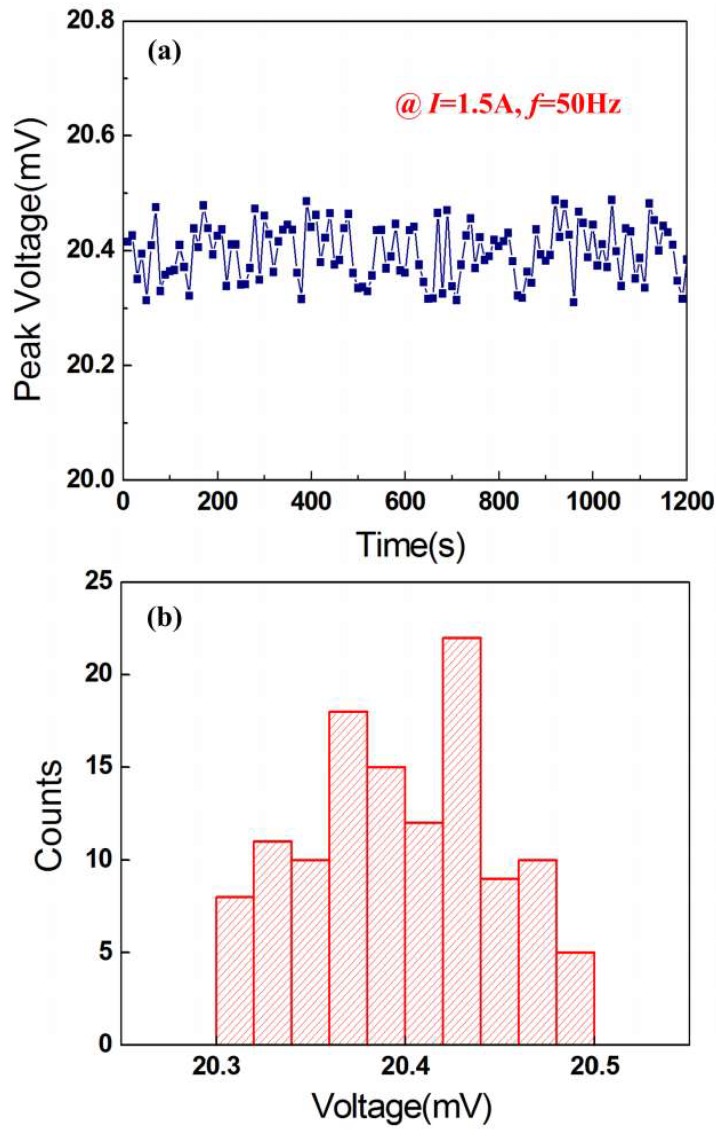
(**a**) Induced voltage versus time at 50 Hz for *d* = 0 mm and (**b**) Histogram for the voltage at 1.5 A.

The current remains unchanged at 1.5 A (*f* = 50 Hz). Figure 5a plots the induced experimental voltage at different times. As can be seen from Figure 5a, the voltage changes with time. The average voltage is 20.4 mA (measurement number *n* = 120) in the given time range (0–1200 s). The histogram of the voltages is shown in Figure 5b. It can be seen from Figure 5b that the voltages approximately obey normal distribution. Therefore, the accuracy of the device for current sensing can be calculated by

(12) $$A=v\pm \frac{4\sigma }{\sqrt{n}},$$

where *ν* is the systematic error, *σ* is the standard deviation, and $\pm 4\sigma /\sqrt{n}$ represents the uncertainty. If we do not take into account the systematic error, the accuracy of the device is calculated to be ±0.0177 mV.

**Figure 6:**
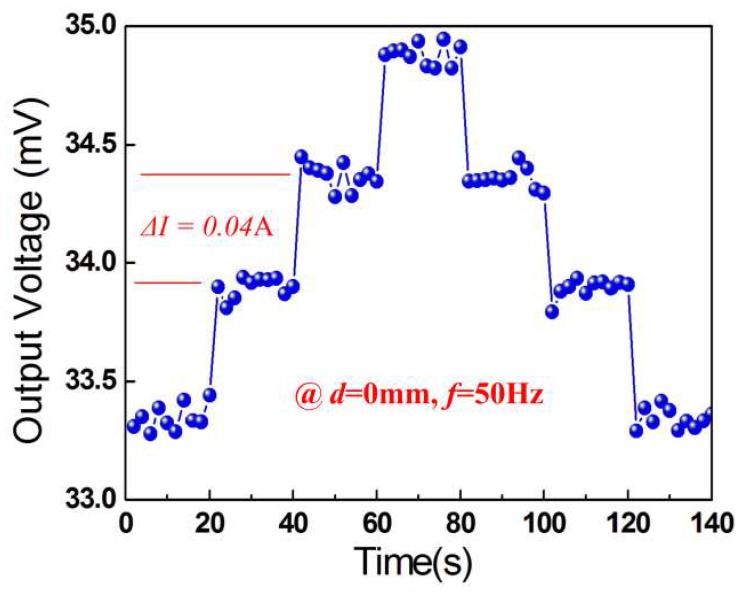
Resolution of the device to the power-frequency current step of 0.04 A at *d* = 0 mm.

A small current step (Δ*I* = 0.04 A, *f* = 50 Hz) was applied in the two-wire power cord. Figure 6 shows the induced experimental voltage versus time for *d* = 0 mm. As can be seen from Figure 6, by adjusting the current amplitudes within 140 s, the output voltage exhibits a step change. It is clear that a current change (Δ*I*) of 0.04 A can be distinguished. We predict further resolution improvement could be achieved by replacing the PZT5H with shear-mode PMN-PT single crystal, which has a higher piezoelectric coefficient *d*_15_.

**Figure 7:**
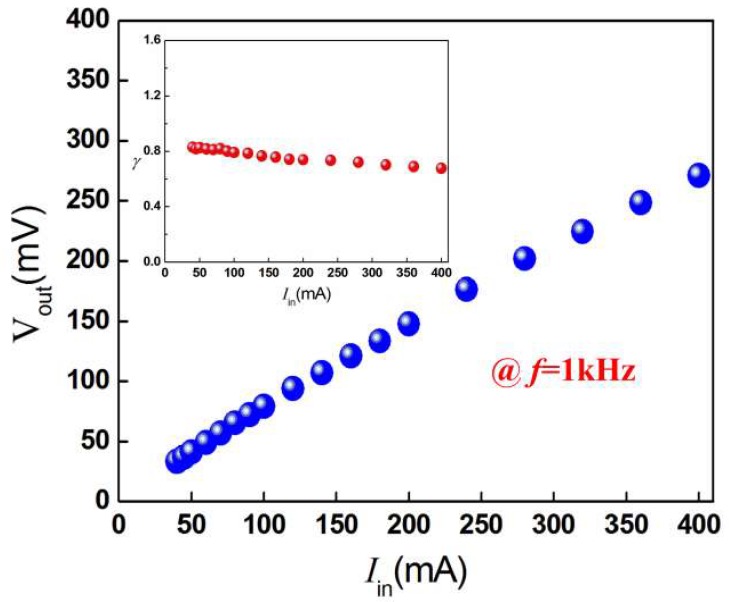
Output open-circuit voltage as a function of the input electric current in the coil at 1 kHz. The inset indicates the conversion factor in the current range of 40 mA to 400 mA.

A copper wire coil was wound around the permalloy plate of the prototype, and a current of 40 mA–400 mA (*I*_in_) was applied in the coil for current-to-voltage conversion. The experimental output peak voltage increases from 33.18 mV to 270.85 mV with approximately linear response to the current at the low-frequency of 1 kHz, as shown in Figure 7. It also can be seen from the inset of Figure 7 that the factor *λ* exhibits an approximate flat response. It varies in the range of 0.68 V/A to 0.83 V/A, with an average value of 0.77 V/A. The results show that the heterostructure has the potential to produce large magnetoelectric effect without using magnetostrictive materials and bias magnetic field [27,28].

## 4. Conclusions

In this paper, a non-resonant piezoelectric current sensing device with high resolution is proposed, which possesses the advantages of passivity, low cost, and simple structure. The device can operate non-invasively for a two-wire power cord carrying opposite currents. The sensitivity of the structure is improved due to the force amplification effect of the cymbal structure and the concentration effect of the permalloy plate. A theoretical model has been developed and validated by the experiments. A large current sensitivity of 12.74 mV/A (*d* = 0 mm) and a high linearity of 0.67% (*d* = 3 mm) are obtained. The experimental current-to-voltage results demonstrate the potential of a large magnetoelectric effect of the proposed piezoelectric device at low-frequency applications.
